# Supplementary material for: Unique ligand and kinase-independent roles of the insulin receptor in regulation of cell cycle, senescence and apoptosis
Source: Nat Commun. 2023 Jan 4;14:57. doi: 10.1038/s41467-022-35693-5 (PMC9812992; doi:10.1038/s41467-022-35693-5)
Supplement: Supplementary file 1 — Supplemental Information [file 41467_2022_35693_MOESM1_ESM.pdf]

Supplemental Materials

Unique Ligand and Kinase-Independent Roles of the Insulin Receptor in Regulation of Cell Cycle, Senescence and Apoptosis

Hirofumi Nagao[1,] Ashok Kumar Jayavelu[2,3], Weikang Cai[1,4], Hui Pan[5], Jonathan M. Dreyfuss[5], Thiago M. Batista[1], Bruna B. Brandão[1], Matthias Mann[2] and C. Ronald Kahn[1*]

[1]Section of Integrative Physiology and Metabolism, Joslin Diabetes Center, Harvard Medical School, Boston, Massachusetts 02215, USA.

[2]Department of Proteomics and Signal Transduction, Max Planck Institute of Biochemistry, 82152 Martinsried, Germany.

[3]Proteomics and Cancer Cell Signaling Group, Clinical Cooperation Unit Pediatric Leukemia, German Cancer Research Center (DKFZ), Heidelberg, Germany.

[4]Department of Biomedical Sciences, New York Institute of Technology College of Osteopathic Medicine, Old Westbury, NY 11568, USA

[5]Bioinformatics and Biostatistics Core, Joslin Diabetes Center, Harvard Medical School, Boston, Massachusetts 02215, USA.

*Correspondence and requests for materials should be addressed to:
C. Ronald Kahn, MD
Joslin Diabetes Center, One Joslin Place, Boston, MA 02215, USA
Phone (617 309-2635)
e-mail: c.ronald.kahn@joslin.harvard.edu

Abbreviations

insulin receptor, IR; ligand and tyrosine kinase-independent, LYK-I; ligand and tyrosine kinase-dependent, LYK-D; phosphatidylinositol 3-kinase, PI3K; IGF-1 receptor, IGF1R; cells lacking both IR and IGF1R, DKO cells; lacking 79 amino acids from the C-terminus, ΔCT; Juxtamembrane-Domain-Only, JMO; T-distributed Stochastic Neighbor Embedding, t-SNE; intracellular domain, ICD; extracellular domain, ECD; extracellular matrix, ECM; Small Ubiquitin-like Modifier, SUMO; promyelocytic leukemia protein, PML; nuclear body, NB; interferon, IFN; interferon-stimulated genes, ISGs; protein phosphatase 1A, PPM1A; protein tyrosine phosphatase type IVA 3, PTP4A3; dual-specificity phosphatases, DUSPs; Principal component analysis, PCA; senescence-associated secretory phenotype, SASP; IFN-stimulated gene factor 3, ISGF3; IFN-stimulated response elements, ISREs.

Supplementary Fig. 1

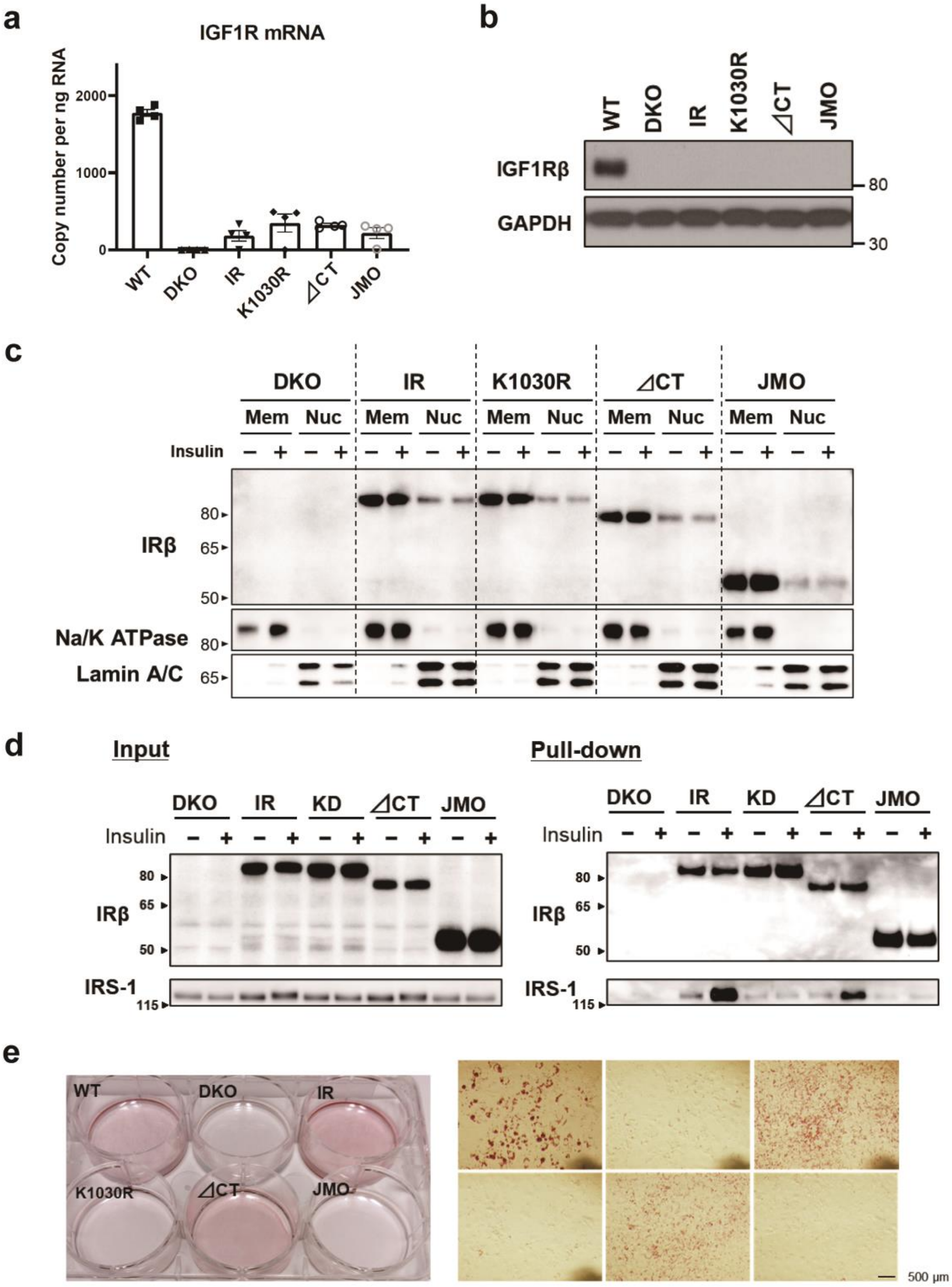

Supplementary Fig. 1. Kinase- and domain-dependent roles of IR in regulating metabolism and growth.

(a) mRNA levels of IGF1R in WT, DKO, IR, K1030R, ΔCT and JMO cells as determined by qPCR using cDNA standards for quantitation. Data are means ± SEM copy number per ng total RNA, n = 4. **(b)** Immunoblotting of IGF1R in lysates from WT, DKO, IR, K1030R, ΔCT and JMO cells. **(c)** Immunoblotting of IRβ in lysates of membrane (Mem) and nucleus (Nuc) fraction from DKO, IR, K1030R, ΔCT and JMO cells. Cells were serum starved 6 h and stimulated with 100 nM insulin for 30 min or left untreated. **(d)** Flag-tagged receptor-containing protein complexes were pulled down with anti-flag magnetic beads following 100 nM insulin stimulation for 15 min and subjected to SDS-PAGE immunoblotting. Bound IR and IRS-1 were detected using anti-IRβ antibody and anti-IRS-1 antibody, respectively. **(e)** Oil red O staining images of WT, DKO, IR, K1030R, ΔCT and JMO cells day 7 after induction of differentiation. Scale bar; 500 µm.

Supplementary Fig. 2

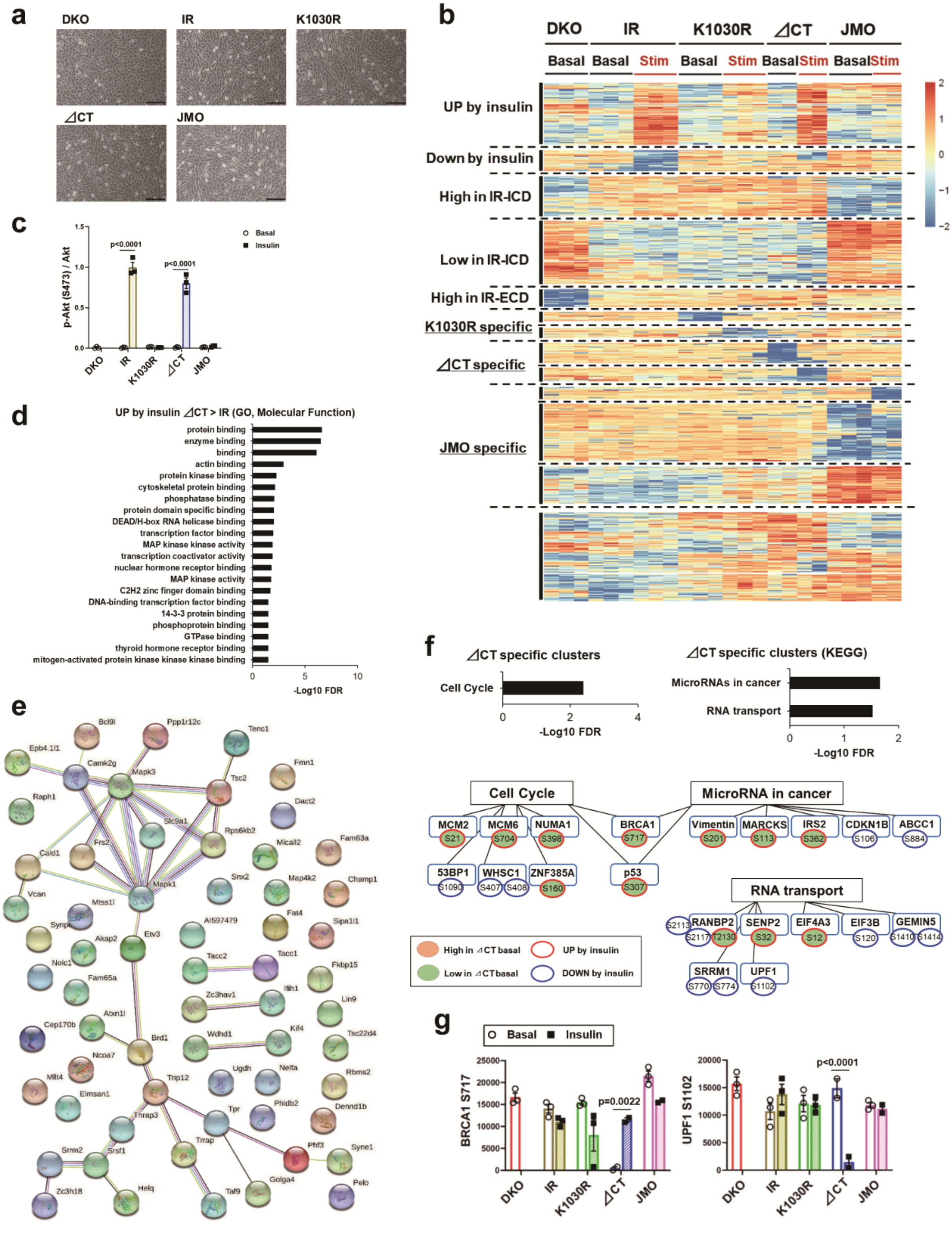

Supplementary Fig. 2. Phosphoproteomic analysis of UP by insulin: ΔCT > IR and ΔCT specific clusters.

(a) Photographs are representative images of DKO, IR, K1030R, ΔCT and JMO cells used for experiments. Scale bar; 200 μm. **(b)** Heatmap showing the hierarchical clustering of the phosphopeptides using phosphopeptides that have at least 80% observed values in all samples. Values are Z-scores of log2 transformed intensity values. **(c)** Densitometric analysis of phosphor-Akt in lysates from DKO, IR, K1030R, ΔCT and JMO cells in the basal state. The level of phosphor-Akt in the insulin stimulated (15 min) IR cells was set at 1. Data are means ± SEM (n = 3 per group). P-values are basal vs insulin stimulation, two-way ANOVA followed by Šídák's multiple comparisons test. **(d)** The top-enriched GO term (Molecular Function) pathways in the UP by insulin: ΔCT > IR cluster. The functional enrichment analysis was tested by the STRING database, where FDRs were calculated using the Benjamini-Hochberg procedure. Plots are –log10 transforms of enrichment FDR value. **(e)** The protein interaction network of the UP by insulin: ΔCT > IR cluster was obtained from the STRING database. **(f)** Enriched REACTOME and KEGG pathways of phosphosites and diagram of intracellular signaling regulated by ΔCT cells in the ΔCT specific clusters. The functional enrichment analyses were tested by the STRING database, where FDRs were calculated using the Benjamini-Hochberg procedure. The top phosphosites that have FDR < 0.05 in the F-tests were selected. Phosphosites were color coded based on the effects of basal or insulin stimulation on phosphorylation. **(g)** Quantification of exemplary phosphosites in the enriched pathways (in Supplementary Fig. 2d) in the ΔCT specific clusters. Data are means ± SEM of phosphosites intensity values ($\times 10^4$) (n = 2-3)**.** P-values are basal vs insulin stimulation, two-way ANOVA followed by Šídák's multiple comparisons test.

Supplementary Fig. 3

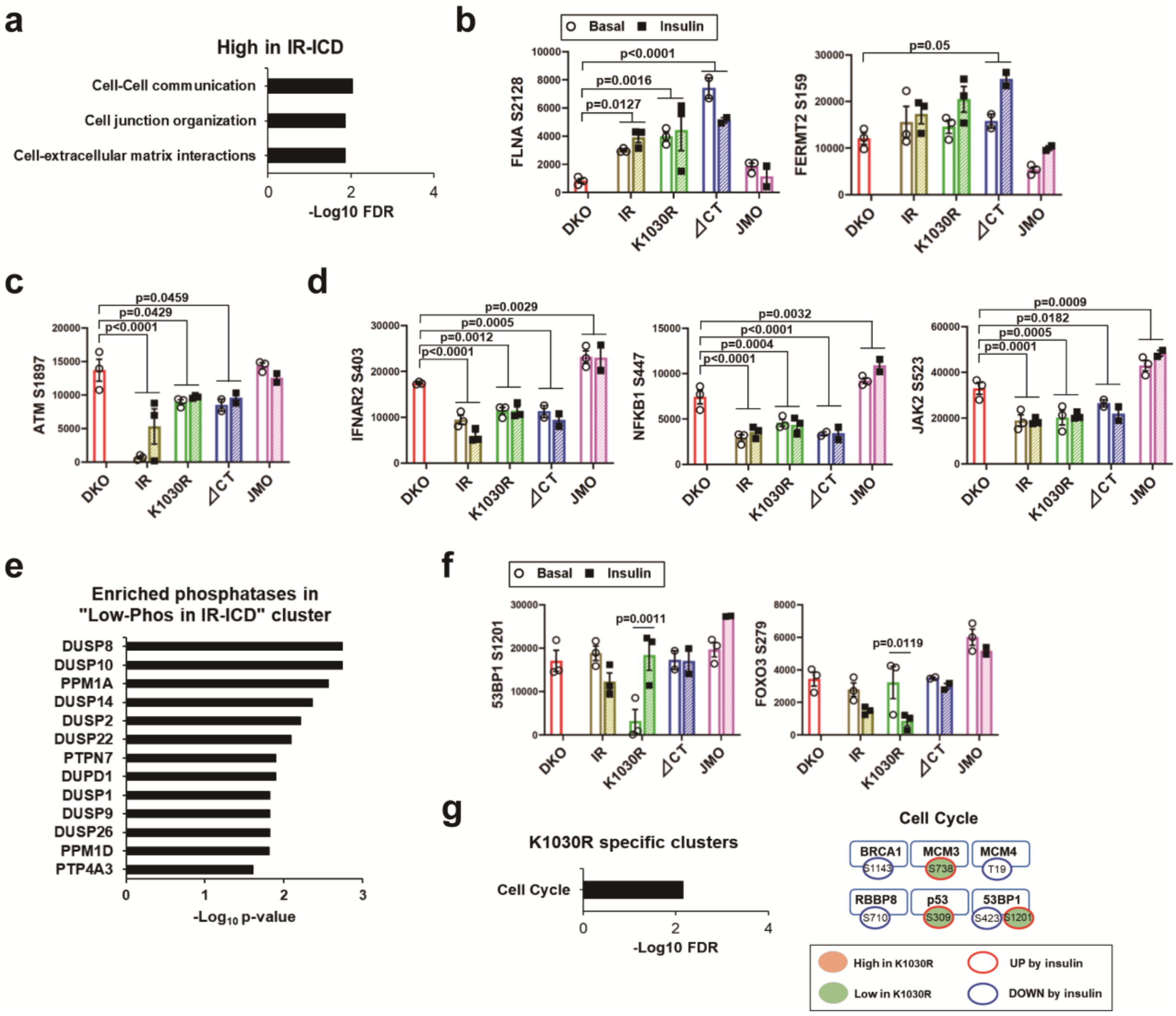

Supplementary Fig. 3. Phosphoproteomic analysis of IR-ICD related clusters and receptor specific signaling regulated by mutated IRs.

(a) REACTOME pathway enrichment analysis of phosphosites in the High-Phos in IR-ICD cluster. The functional enrichment analysis was tested by the STRING database, where FDRs were calculated using the Benjamini-Hochberg procedure. Plots are –log10 transforms of enrichment FDR value. **(b)** Quantitation of exemplary phosphosites in the enriched pathways (in Supplementary Fig. 3a) in the High-Phos in IR-ICD cluster. Data are means ± SEM of phosphosite intensity values ($\times 10^4$). P-values vs DKO (combined both basal and insulin for comparisons), one-way ANOVA followed by Dunnett's multiple comparisons test (n = 3-6). **(c)** Quantitation of phosphorylation in ATM^{S1897}. Data are means ± SEM of phosphosite intensity values ($\times 10^4$). P-values vs DKO, one-way ANOVA (n = 3-6). **(d)** Quantification of some cellular senescence signaling associated phosphosites in the Low-Phos in IR-ICD cluster. Data are means ± SEM of phosphosite intensity values ($\times 10^4$). P-values vs DKO, one-way ANOVA (n = 3-6). **(e)** Phosphatase enrichment analysis for phosphosites in the Low-Phos in IR-ICD cluster by using the DEPOD (DEPhOsphorylation Database). Enriched phosphatases were tested using the Fisher exact test. Plots are –log10 transforms of enrichment P value. **(f)** Quantification of exemplary phosphosites in the K1030R specific clusters in Fig. 2c. Data are means ± SEM of phosphosites intensity values ($\times 10^4$) (n = 2-3). P-values are basal vs insulin stimulation, two-way ANOVA followed by Šídák's multiple comparisons test. **(g)** REACTOME pathway enrichment analysis of phosphosites in the K1030R specific clusters. The functional enrichment analysis was tested by the STRING database, where FDRs were calculated using the Benjamini-Hochberg procedure. Diagram of intracellular signaling regulated by K1030R as identified by phosphoproteomics. The top phosphosites that have FDR < 0.05 in the F-tests were selected. Phosphosites were color coded based on the effects of basal or insulin stimulation on phosphorylation.

Supplementary Fig. 4

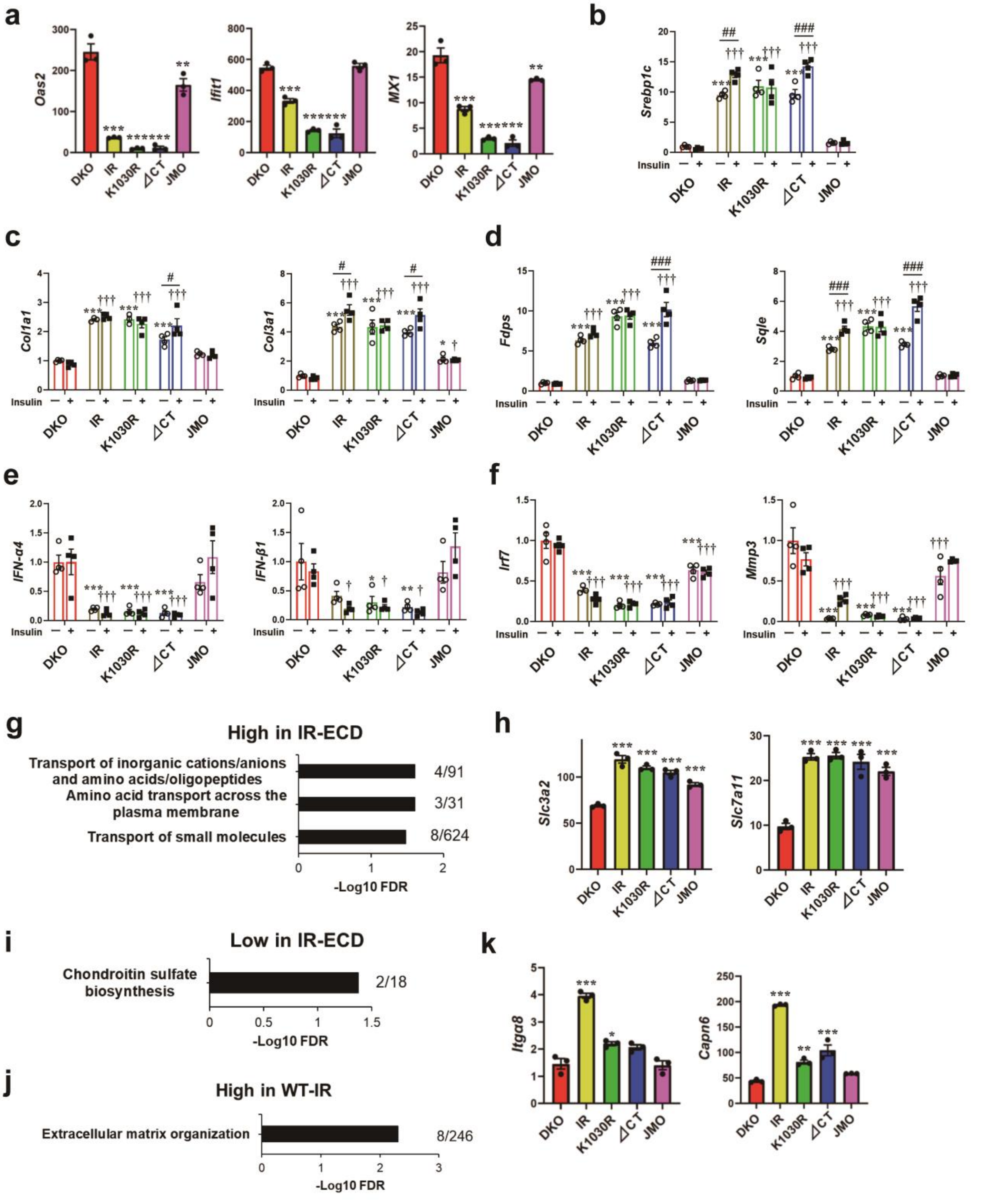

Supplementary Fig. 4. Differential regulated mRNA patterns by DKO, IR, K1030R, ΔCT and JMO cells.

(a) Downstream gene expressions of STAT1/2-ISGs signaling in DKO, IR, K1030R, ΔCT and JMO cells at the basal. Data are means ± SEM (n = 3). ** P < 0.01, *** P < 0.001 vs DKO, one-way ANOVA. **(b-f)** Cells were FBS starved for 5 h with DMEM containing 0.1% BSA, then stimulated with or without 100 nM insulin for 6 h. mRNA levels of genes were analyzed by qPCR. **(b)** mRNA levels of *Srebp1c* in DKO, IR, K1030R, ΔCT and JMO cells. **(c and d)** mRNA levels of collagens **(c)** and genes related cholesterol biosynthesis **(d)** in the High Expression in IR-ICD in Fig.5. **(e and f)** mRNA levels of interferons **(e)** and genes related interferon signaling and SASP **(f)** in Fig.5. Data in **(b-f)** are means ± SEM (n = 4 per group). Gene expression levels of DKO cells at the basal were set at 1. * P < 0.05, ** P < 0.01, *** P < 0.001 vs DKO basal, † P < 0.05, ††† P < 0.001 vs DKO with insulin, # P < 0.05, ## P < 0.01, ### P < 0.001 basal vs insulin, two-way ANOVA. TBP expression was used to normalize gene expression. The sequences of primers as follows: mouse Srebp1c, 5'-GAGCCATGGATTGCACATTT-3' and 5'-CTCAGGAGAGTTGGCACCTG-3'; mouse Fdps, 5'-AGCCGAAGAAACAGGATGCTGAGA-3' and 5'-TTCCAGAAGCAGAGCGTCGTTGAT-3'; mouse Sqle, 5'-TTGTTGCGGATGGACTCTTCTCCA-3' and 5'-GTTGACCAGAACAAGCTCCGCAAA-3'; mouse Col1a1, 5'- GTCCCAACCCCCAAAGAC-3' and 5'-CAGCTTCTGAGTTTGGTGATA-3'; mouse Col3a1, 5'- TGGTTTCTTCTCACCCTTCTTC-3' and 5'-TGCATCCCAATTCATCTACGT-3'; mouse Irf7, 5'-GAGACTGGCTATTGGGGGAG-3' and 5'-GACCGAAATGCTTCCAGGG-3'; mouse Mmp3, 5'-ACATGGAGACTTTGTCCCTTTTG-3' and 5'- TTGGCTGAGTGGTAGAGTCCC-3'. **(f)** Enriched REACTOME pathways of genes in the High Expression in IR-ECD cluster. Plots are –log10 transforms of enrichment FDR value. **(g)** Quantification of exemplary genes in the High Expression in IR-ECD cluster at the basal. **(h)** Enriched REACTOME pathways of genes in the Low Expression in IR-ECD cluster. **(i)** Enriched REACTOME pathways of genes in the High Expression in WT-IR cluster. **(j)** Quantification of exemplary genes in the High Expression in WT-IR cluster at the basal. Data in (a, h and k) are means ± SEM of genes intensity values. * P < 0.05, ** P < 0.01, *** P < 0.001 vs DKO, one-way ANOVA. The functional enrichment analyses in (g, i and j) was tested by the STRING database, where FDRs were calculated using the Benjamini-Hochberg procedure.

Supplementary Fig. 5

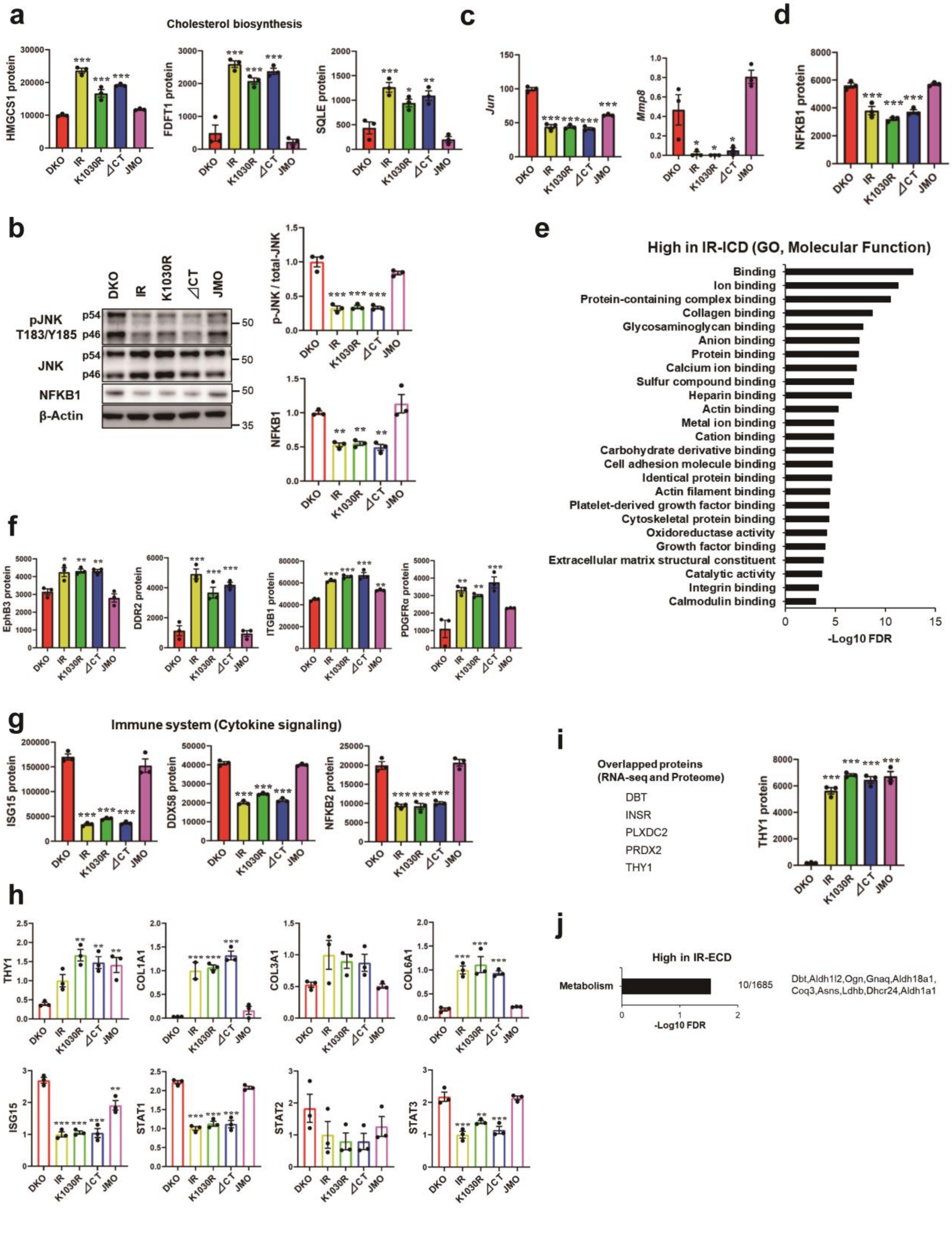

Supplementary Fig. 5. Differential regulated protein patterns by DKO, IR, K1030R, ΔCT and JMO cells.

(a) Quantification of exemplary proteins of cholesterol biosynthesis from the High Protein Expression in IR-ICD cluster at the basal. Data are mean ± SEM of protein intensity values (n = 3). **(b)** Immunoblotting of $JNK^{T183/Y185}$ phosphorylation and NFKB1 in lysates from DKO, IR, K1030R, ΔCT and JMO cells in the basal state. The level of each protein in DKO cells was set at 1. Data are mean ± SEM (n = 3). **(c)** The mRNA levels of *Jun* and *Mmp8*, downstream genes of JNK signaling pathway, in the RNA-seq. Data are means ± SEM of genes intensity values (n = 3). **(d)** The protein levels of NFKB1 in the proteome. Data are means ± SEM of protein intensity values (n = 3). **(e)** The top 25 Gene Ontology (GO) pathways of Molecular Function in the High Protein Expression in IR-ICD cluster. The functional enrichment analysis was tested by the STRING database, where FDRs were calculated using the Benjamini-Hochberg procedure. Plots are –log10 transforms of enrichment FDR value. **(f)** Quantification of exemplary proteins of signaling receptors upregulated by IR-ICD. All proteins except PDGFRα (in the High Expression in IR-ECD cluster) are in the High Expression in IR-ICD cluster. Data are mean ± SEM of protein intensity values (n = 3). **(g)** Quantification of exemplary proteins of immune system from the Low Protein Expression in IR-ICD cluster at the basal. Data are mean ± SEM of protein intensity values (n = 3). **(h)** Densitometric analysis of proteins in Fig. 6i in lysates from DKO, IR, K1030R, ΔCT and JMO cells in the basal state. The level of each protein in IR cells was set at 1. Data are means ± SEM (n = 3). **(i)** Overlapped proteins which showed same changes in the RNA-seq and the proteome, and quantification of exemplary protein in the High Protein Expression in IR-ECD cluster at the basal. Data are means ± SEM of protein intensity values (n = 3). **(j)** Enrichment analysis of REACTOME pathway of proteins in the High Protein Expression in IR-ECD cluster. The functional enrichment analysis was tested by the STRING database, where FDRs were calculated using the Benjamini-Hochberg procedure. Plots are –log10 transforms of enrichment FDR value. The proteins in the “Metabolism” pathway are also shown. All data in Supplementary Fig. 5 are * $P < 0.05$, ** $P < 0.01$, *** $P < 0.001$ vs DKO, one-way ANOVA.

Supplementary Fig. 6

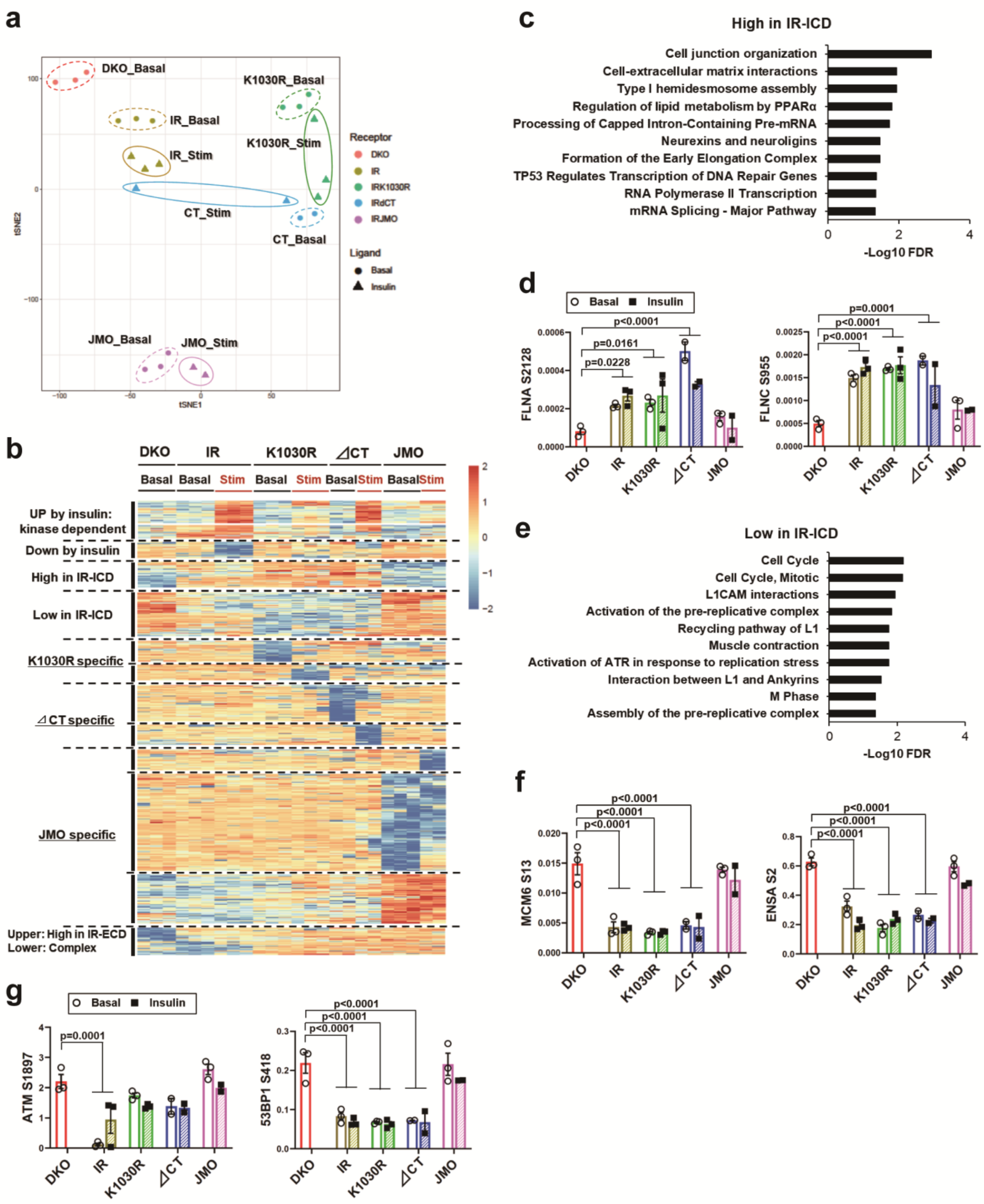

Supplementary Fig. 6. Differential phosphoproteomic signatures (normalized by the proteome data) of DKO, IR, K1030R, ΔCT and JMO cells.

(a) T-distributed stochastic neighbor embedding (t-SNE) analysis of the phosphosites identified by LC-MS/MS from DKO, IR, K1030R, ΔCT and JMO cells in the basal and insulin stimulated states. Phosphosites intensity values were divided by each protein intensity value in the proteome data. **(b)** Heatmap showing the hierarchical clustering of the phosphopeptides in DKO, IR, K1030R, ΔCT and JMO cells in the basal and insulin stimulated states. Values are Z-scores of log2 transformed intensity values. **(c)** REACTOME pathway enrichment analysis of phosphosites in the High-Phos in IR-ICD cluster. The functional enrichment analysis was tested by the STRING database, where FDRs were calculated using the Benjamini-Hochberg procedure. Plots are –log10 transforms of enrichment FDR value. **(d)** Quantitation of exemplary phosphosites in the enriched pathways (in Supplementary Fig. 6c) in the High-Phos in IR-ICD cluster. Data are means ± SEM of phosphosites intensity values divided by each protein intensity value (n = 3-6). P-values vs DKO (combined both basal and insulin for comparisons), one-way ANOVA followed by Dunnett's multiple comparisons test. **(e)** REACTOME pathway enrichment analysis of phosphosites in the Low-Phos in IR-ICD cluster. The functional enrichment analysis was tested by the STRING database, where FDRs were calculated using the Benjamini-Hochberg procedure. **(f)** Quantitation of exemplary phosphosites in the enriched pathways (in Supplementary Fig. 6e) in the Low-Phos in IR-ICD cluster. Data are means ± SEM of phosphosites intensity values divided by each protein intensity value (n = 3-6). P-values vs DKO, one-way ANOVA. **(g)** Some important phosphosites normalized by the proteome data in the ATM signaling pathway. Data are means ± SEM of phosphosites intensity values divided by each protein intensity value (n = 3-6). P-values vs DKO, one-way ANOVA.

Supplementary Fig. 7

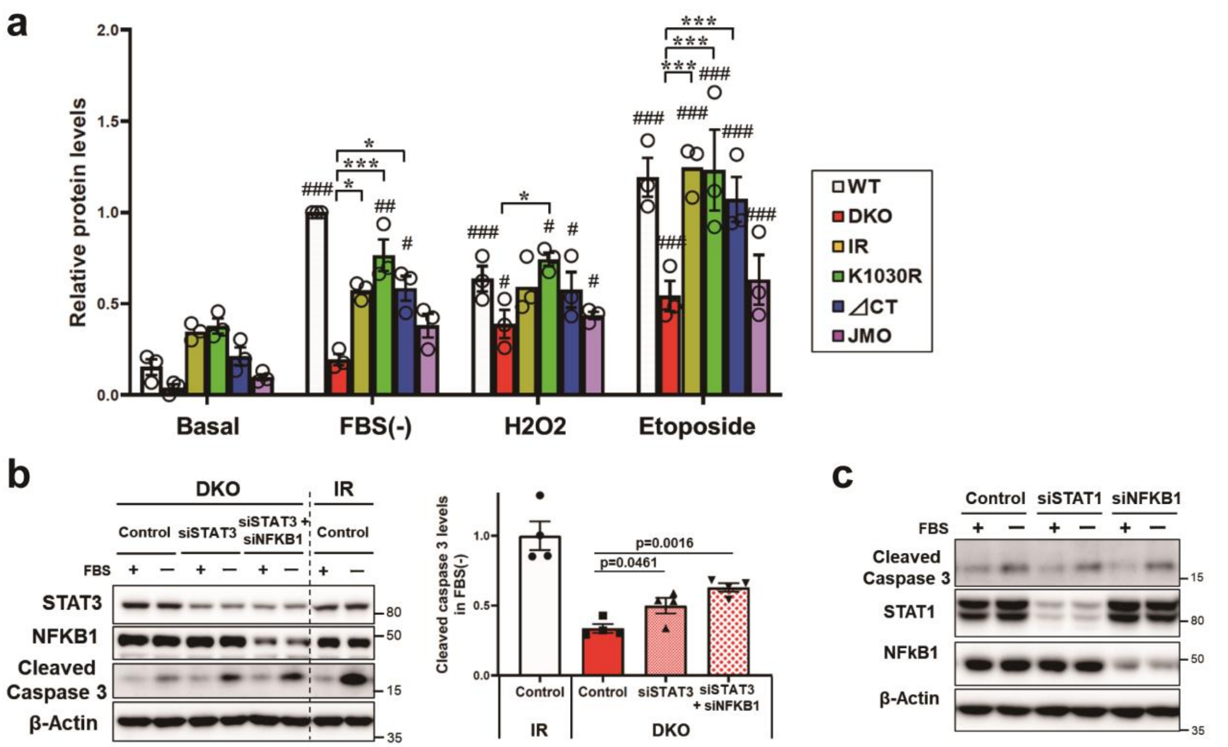

Supplementary Fig. 7. Changes in apoptotic sensitivity in DKO, IR, K1030R, ΔCT and JMO cells.

(a) Densitometric analysis of cleaved caspase 3 in Fig. 8b in lysates from WT, DKO, IR, K1030R, ΔCT and JMO cells. The level in IR cells with FBS starvation for 6 h was set at 1. Data are means ± SEM (n = 3 per group). * $P < 0.05$, *** $P < 0.001$ vs DKO, # $P < 0.05$, ## $P < 0.01$, ### $P < 0.001$ vs basal, two-way ANOVA. **(b)** Knockdown of STAT3 or both STAT3 and NFKB1 in DKO cells. Immunoblotting of cleaved caspase 3 levels in lysate from DKO and IR cells with or without 6 h FBS starvation (n=4 per group). The level in the IR control was set at 1. Data are means ± SEM. Comparisons between groups was performed using one-way ANOVA. **(c)** Knockdown of STAT1 or NFKB1 in DKO cells. Immunoblotting of cleaved caspase 3 levels in lysate from DKO cells with or without 6 h FBS starvation.

Supplementary Fig. 8

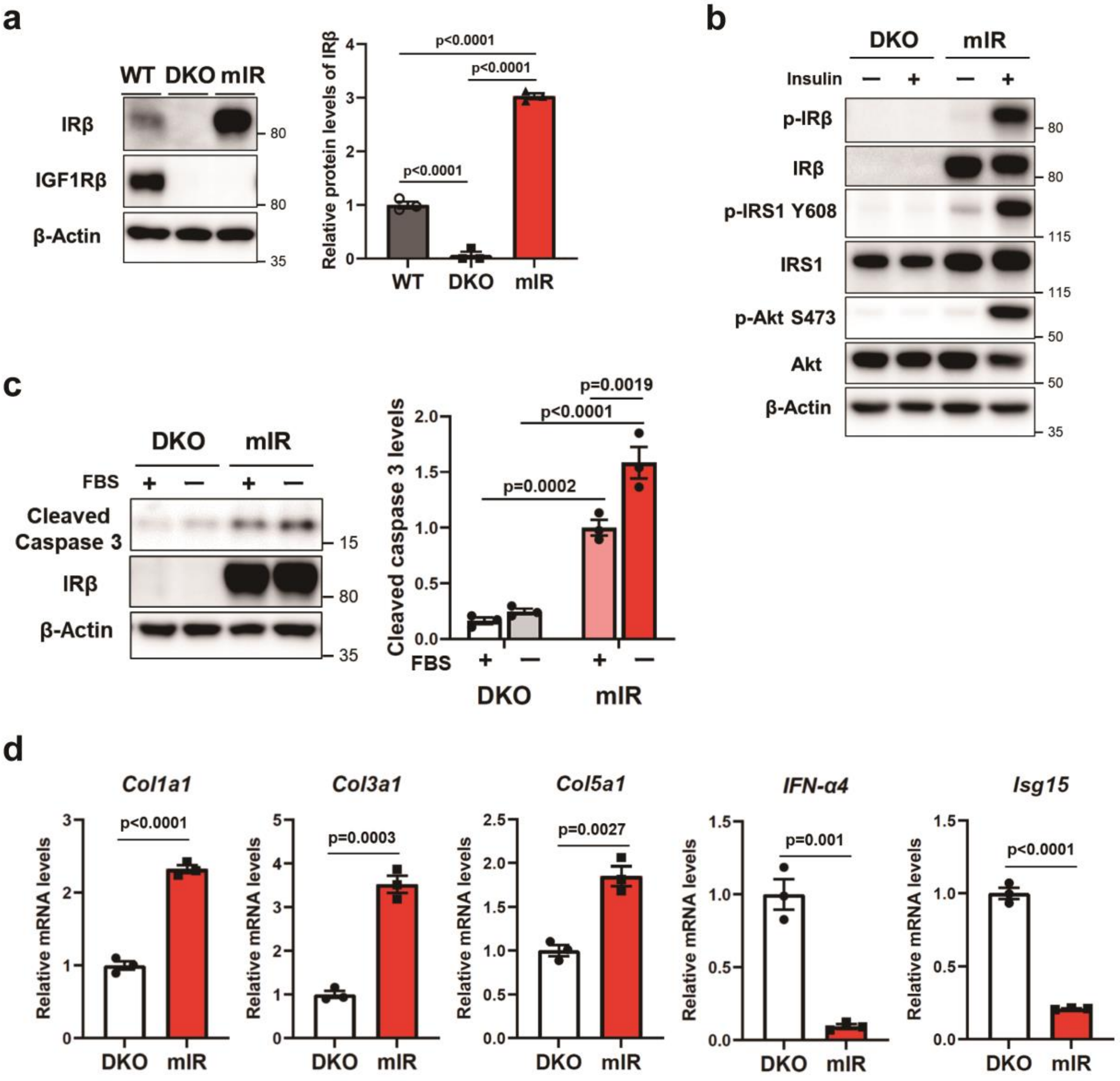

Supplementary Fig. 8. Changes in apoptotic sensitivity and gene expressions in DKO and mouse IR cells.

(a) Immunoblot analysis of WT preadipocytes, DKO preadipocytes and DKO preadipocytes reconstituted with mouse IR (mIR, A isoform) for IR-beta subunit (IRβ), IGF1R-beta subunit (IGF1Rβ) and β-actin, and quantification of IRβ. Data are means ± SEM (n = 3). Comparisons between groups was performed using one-way ANOVA followed by Tukey's multiple comparisons test. **(b)** Immunoblotting of IRS-1^{Y608} phosphorylation and Akt^{S473} phosphorylation in lysates from DKO and mIR cells stimulated with 100 nM insulin for 15 min. **(c)** Immunoblotting of cleaved caspase 3 in DKO and mIR cells following FBS starvation for 6h. For densitometric analysis of cleaved caspase 3, the level in mIR cells without FBS starvation was set at 1. Data are means ± SEM (n = 3). Comparisons between groups was performed using two-way ANOVA. (d) mRNA levels of collagens and genes associated with interferon signaling. Data are means ± SEM (n = 3). Statistical significance was assessed using two-tailed unpaired Student's *t*-test. 36B4 expression was used to normalize gene expression. The sequences of additional primers as follows: mouse Col5a1, 5'-CTTCGCCGCTACTCCTGTTC-3' and 5'- CCCTGAGGGCAAATTGTGAAAA-3'; mouse Isg15, 5'-GGTGTCCGTGACTAACTCCAT-3' and 5'-CTGTACCACTAGCATCACTGTG-3'; mouse 36B4, 5'-GAAACTGCTGCCTCACATCCG-3' and 5'-GCTGGCACAGTGACCTCACACG-3'.

Supplementary Table 1. All IR peptide identified in the proteome dataset

DKO Basal	IR basal	IR Insulin	ΔCT Basal	ΔCT Insulin	JMO Basal	JMO Insulin	K1030R Basal	K1030R Insulin	Sequence
NaN	27,295	2,738,039	NaN	NaN	2,879,249	2,859,987	NaN	NaN	ADDIVGPVTHEIFENNVVHLMWQEPK
NaN	2,442,105	NaN	NaN	NaN	2,586,423	252,158	2,402,251	NaN	CSVAAYVSAR
NaN	2,639,367	2,607,276	2,606,815	2,610,586	2,796,697	274,453	2,602,342	2,630,516	DLFPNLTVIR
NaN	NaN	NaN	2,528,701	247,571	2,685,712	2,707,098	2,529,625	2,511,041	ELEESSFR
NaN	2,507,909	2,540,038	2,507,304	NaN	2,686,077	2,710,129	2,489,422	2,528,849	ESLVISGLR
NaN	2,550,634	2,571,772	2,458,469	2,488,175	NaN	NaN	2,484,249	2,525,911	GFTCHHVVR
NaN	2,562,589	251,437	2,455,516	2,481,973	261,928	2,642,782	245,245	2,499,993	ILDSVEDNYIVLNK
NaN	NaN	NaN	2,550,815	NaN	NaN	NaN	NaN	2,405,747	KHFALER
NaN	2,674,214	2,653,017	2,588,045	2,603,865	2,853,697	2,809,336	2,608,282	2,616,244	LCLSEIHK
NaN	2,676,724	2,683,702	2,621,577	2,596,499	277,026	277,145	2,614,938	2,669,325	LFFHYNPK
NaN	2,618,502	2,653,782	NaN	NaN	2,682,505	NaN	2,648,827	NaN	LIMITDYLLLFR
NaN	239,094	2,422,913	2,377,679	2,407,512	NaN	NaN	NaN	NaN	MCWQFNPK
NaN	NaN	2,477,212	2,575,078	2,496,224	256,446	2,664,083	NaN	NaN	MEEVSGTK
NaN	NaN	2,638,537	2,546,098	2,572,435	NaN	NaN	2,532,093	2,540,484	NCMVAHDFTVK
NaN	NaN	2,301,037	NaN	2,137,082	2,565,696	2,527,465	NaN	NaN	NNELCYLATIDWSR
NaN	2,445,932	2,422,339	NaN	NaN	NaN	NaN	NaN	NaN	PWTQYAIFVK
NaN	NaN	NaN	NaN	NaN	252,303	2,536,713	NaN	NaN	RYGDEELHLCVSR
NaN	2,709,668	272,102	265,497	2,644,398	2,913,075	287,466	2,658,118	2,694,871	TFEDYLHNVVFVPR
NaN	2,636,346	2,605,883	2,489,125	2,506,505	2,727,195	2,693,089	2,557,248	2,586,323	TIDSVTSAQELR
NaN	2,594,315	2,586,084	2,535,171	NaN	2,738,533	2,739,468	2,571,088	2,570,618	TLVTFSDER
NaN	2,606,194	255,389	2,529,463	2,489,514	2,785,079	27,202	2,478,965	2,477,249	TNCPATVINGQFVER
NaN	NaN	NaN	NaN	NaN	2,549,614	2,535,695	NaN	NaN	TNGDQASCENELLK
NaN	2,555,491	2,467,476	2,503,814	2,520,307	265,796	2,620,359	246,473	2,505,904	TRPEDFR
NaN	NaN	2,617,106	2,591,846	2,556,549	NaN	NaN	2,590,355	2,567,239	TVNESASLR
NaN	NaN	2,564,955	245,528	2,528,715	2,679,566	2,739,526	2,523,396	2,484,797	VCPTICK
NaN	2,459,701	2,464,746	234,302	NaN	2,597,926	2,623,513	2,338,811	2,378,324	VYGLESLK
NaN	2,551,803	2,570,294	2,521,111	2,567,206	2,752,828	2,731,121	2,464,477	250,077	YGDEELHLCVSR

All INSR peptide intensities in DKO, IR, K1030R, ΔCT and JMO cells in the proteome dataset. Each intensity value is average of each condition. NaN; Not a Number. It means no detection in the dataset. IGF1R were not detected in all cell lines in the proteomic dataset.
