# Supplementary material for: Unique ligand and kinase-independent roles of the insulin receptor in regulation of cell cycle, senescence and apoptosis
Source: Nat Commun. 2023 Jan 4;14:57. doi: 10.1038/s41467-022-35693-5 (PMC9812992; doi:10.1038/s41467-022-35693-5)
Supplement: Supplementary file 3 — Description of Additional Supplementary Information [file 41467_2022_35693_MOESM3_ESM.pdf]

Description of Additional Supplementary Information

Supplementary Data 1: The phosphoproteome datasheets

Supplementary Data 2: The RNA-seq datasheets

Supplementary Data 3: The proteome datasheets
