# Supplementary material for: Unique ligand and kinase-independent roles of the insulin receptor in regulation of cell cycle, senescence and apoptosis
Source: Nat Commun. 2023 Jan 4;14:57. doi: 10.1038/s41467-022-35693-5 (PMC9812992; doi:10.1038/s41467-022-35693-5)

Original blots related to Figure 1c

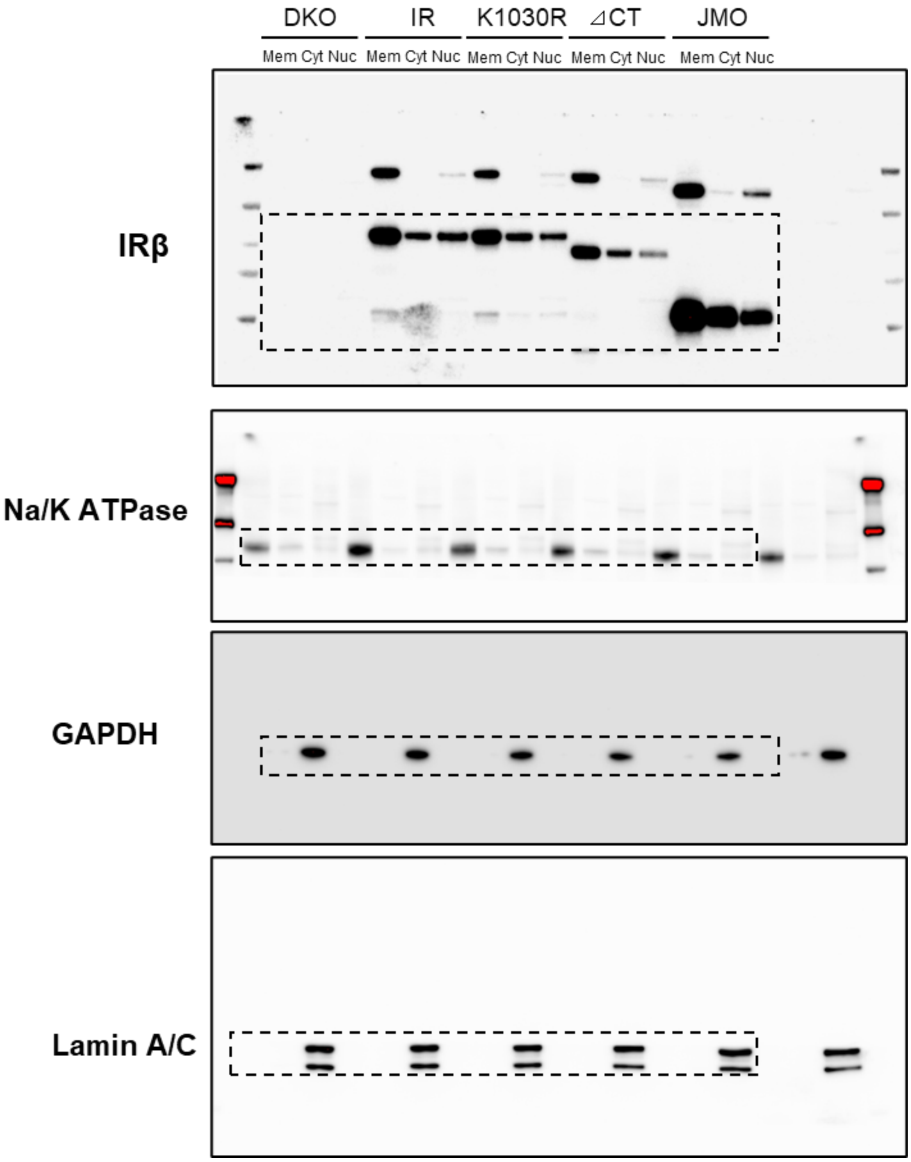

Original blots related to Figure 1d

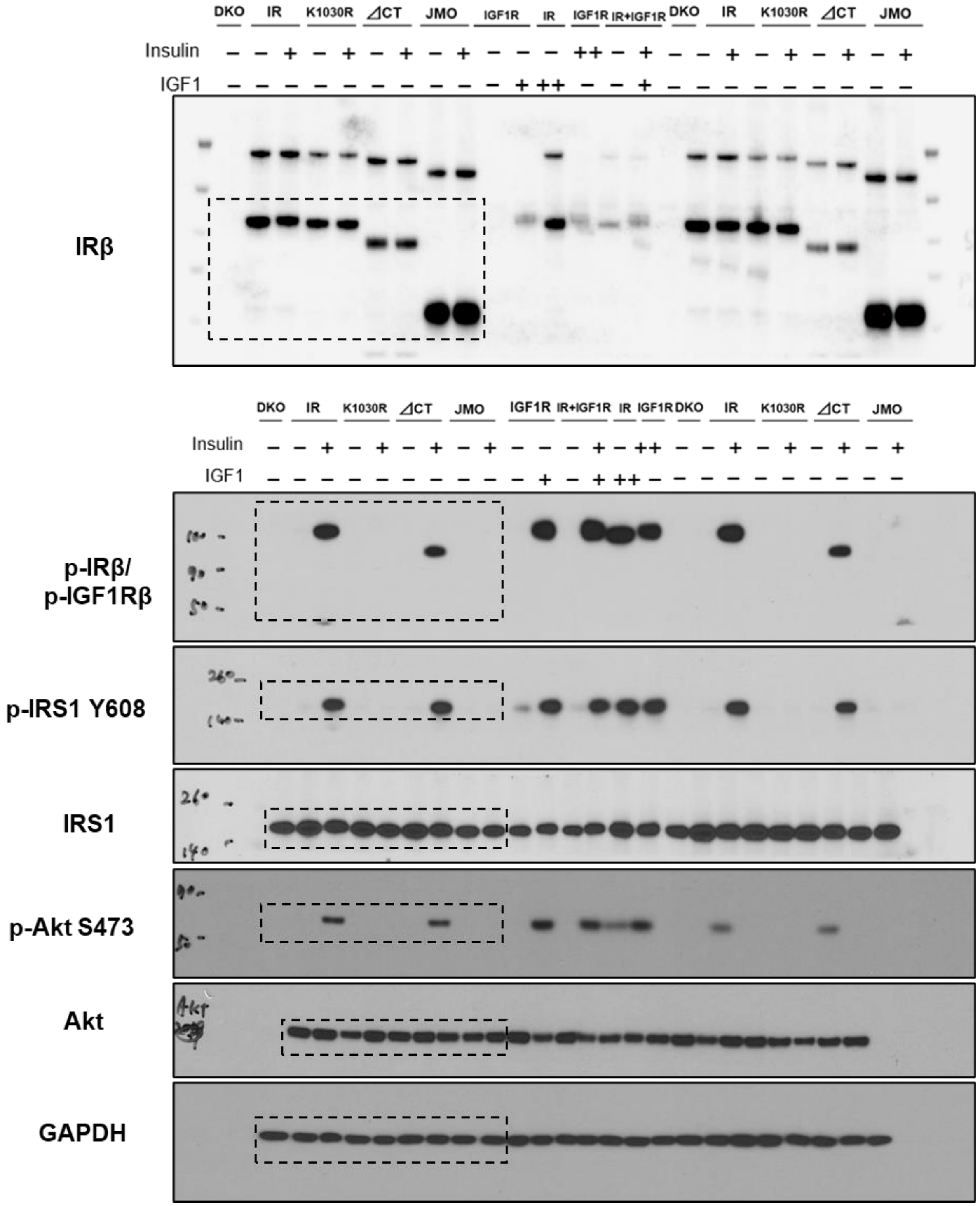

Original blots related to Figure 6j

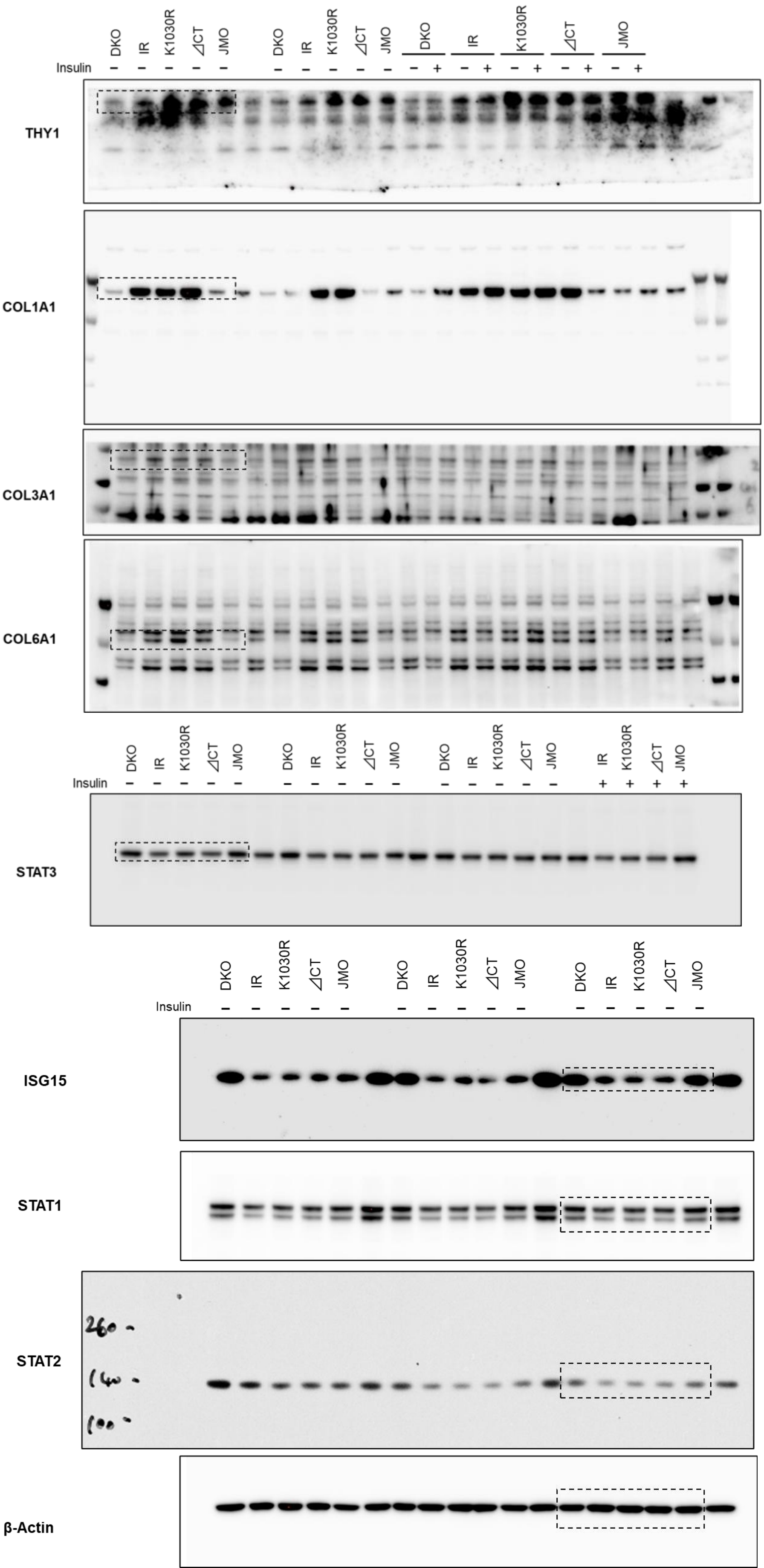

Original blots related to Figure 8b

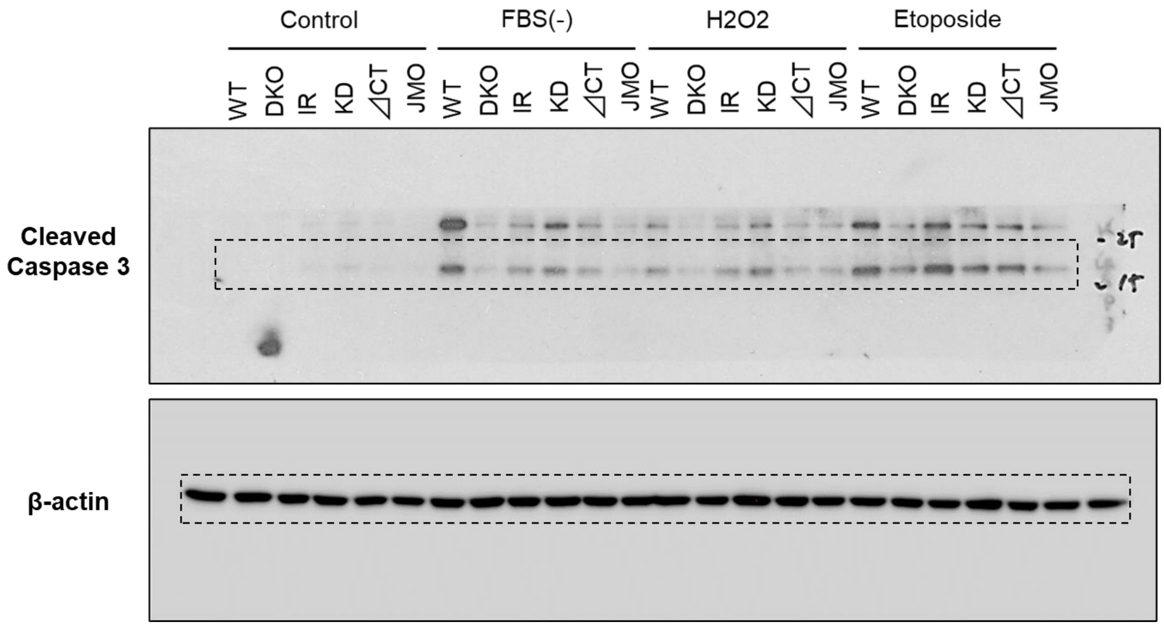

Original blots related to Figure 8d

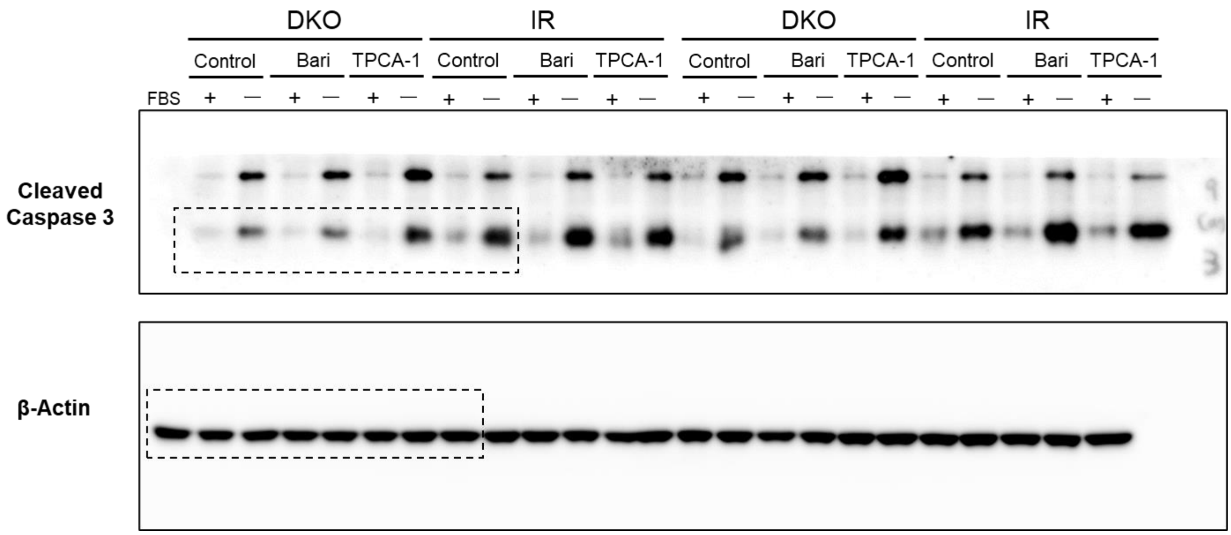

Original blots related to Supplementary Figure 1b

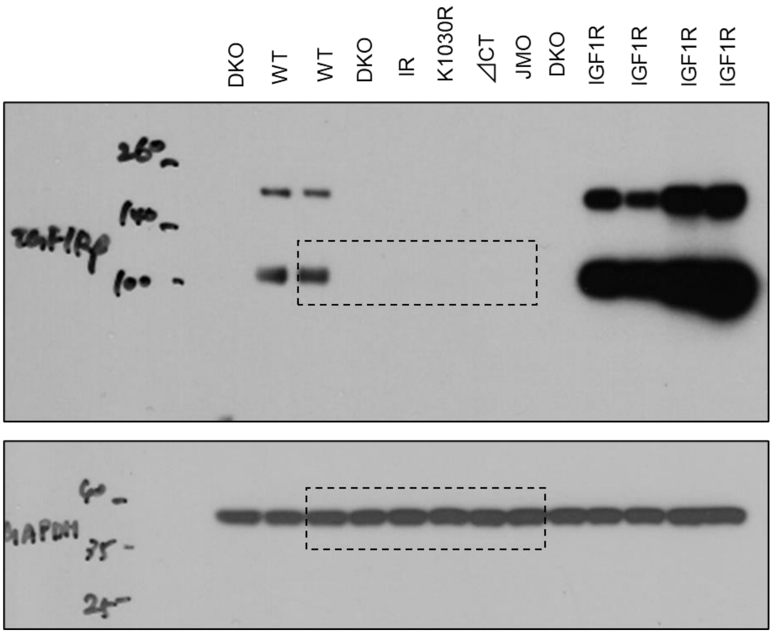

Original blots related to Supplementary Figure 1c

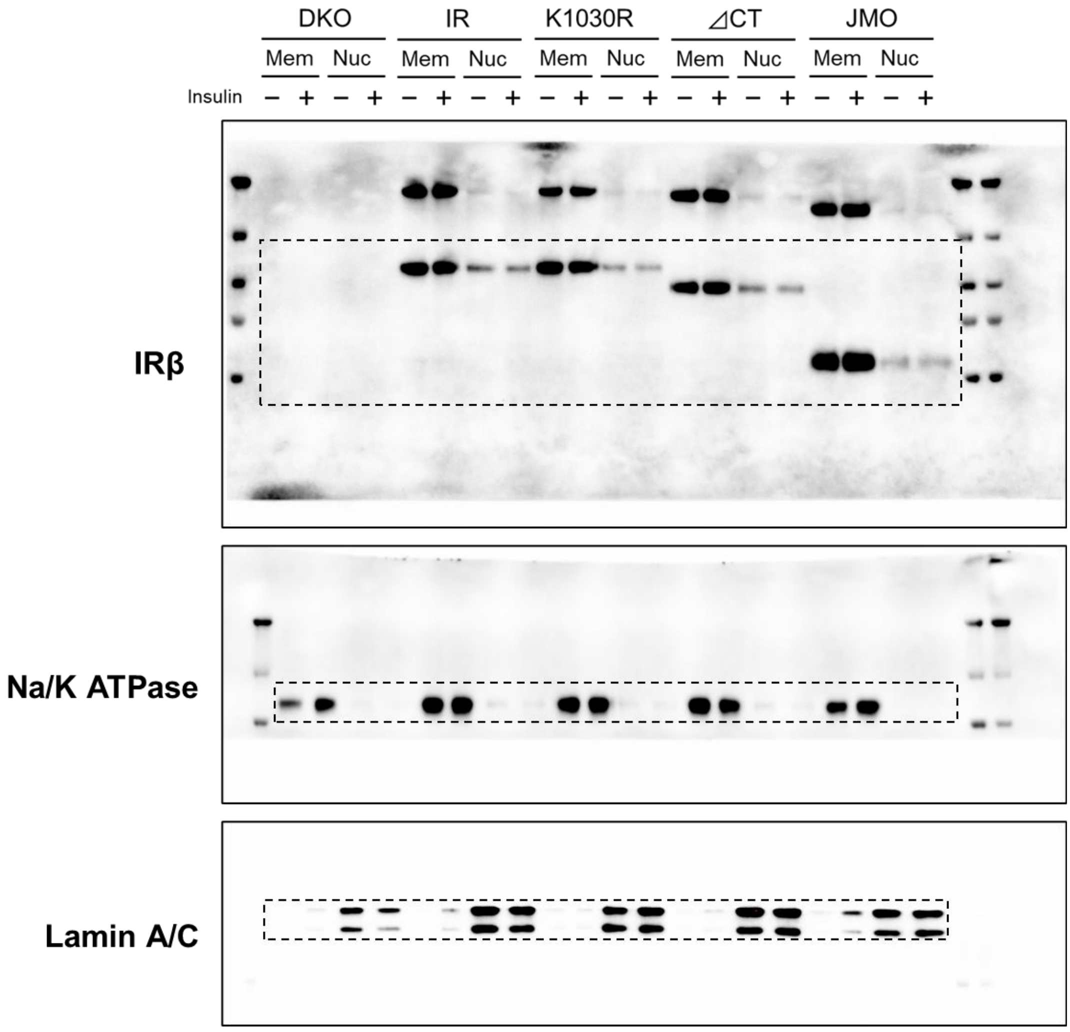

Original blots related to Supplementary Figure 1d

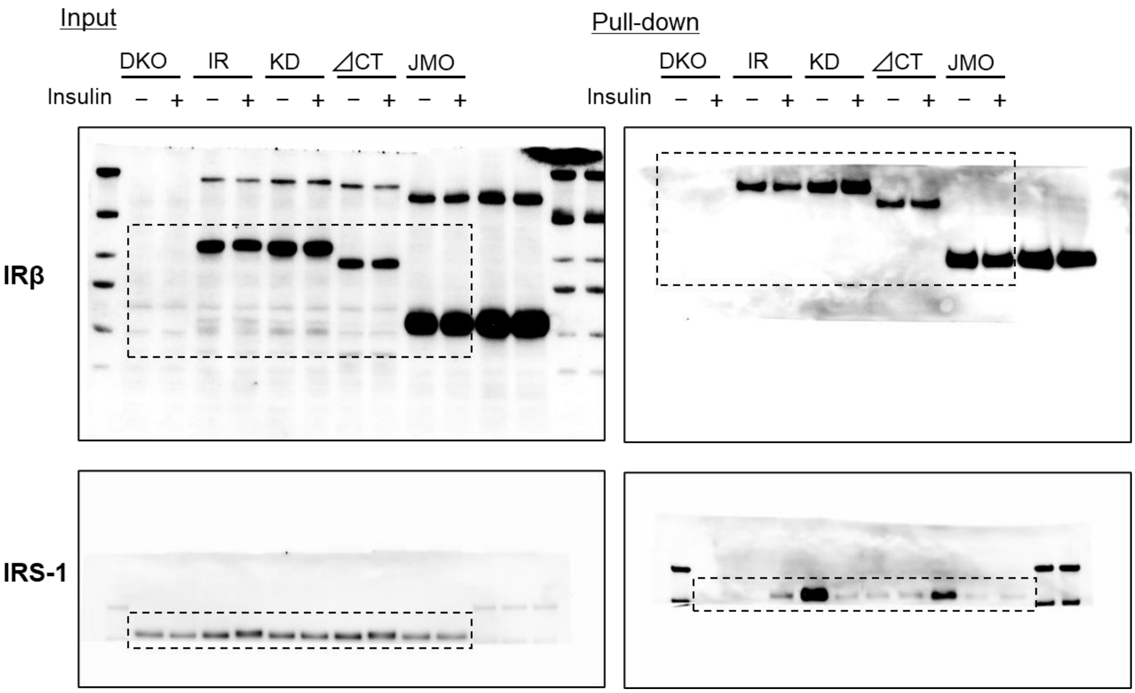

Original blots related to Supplementary Figure 5d

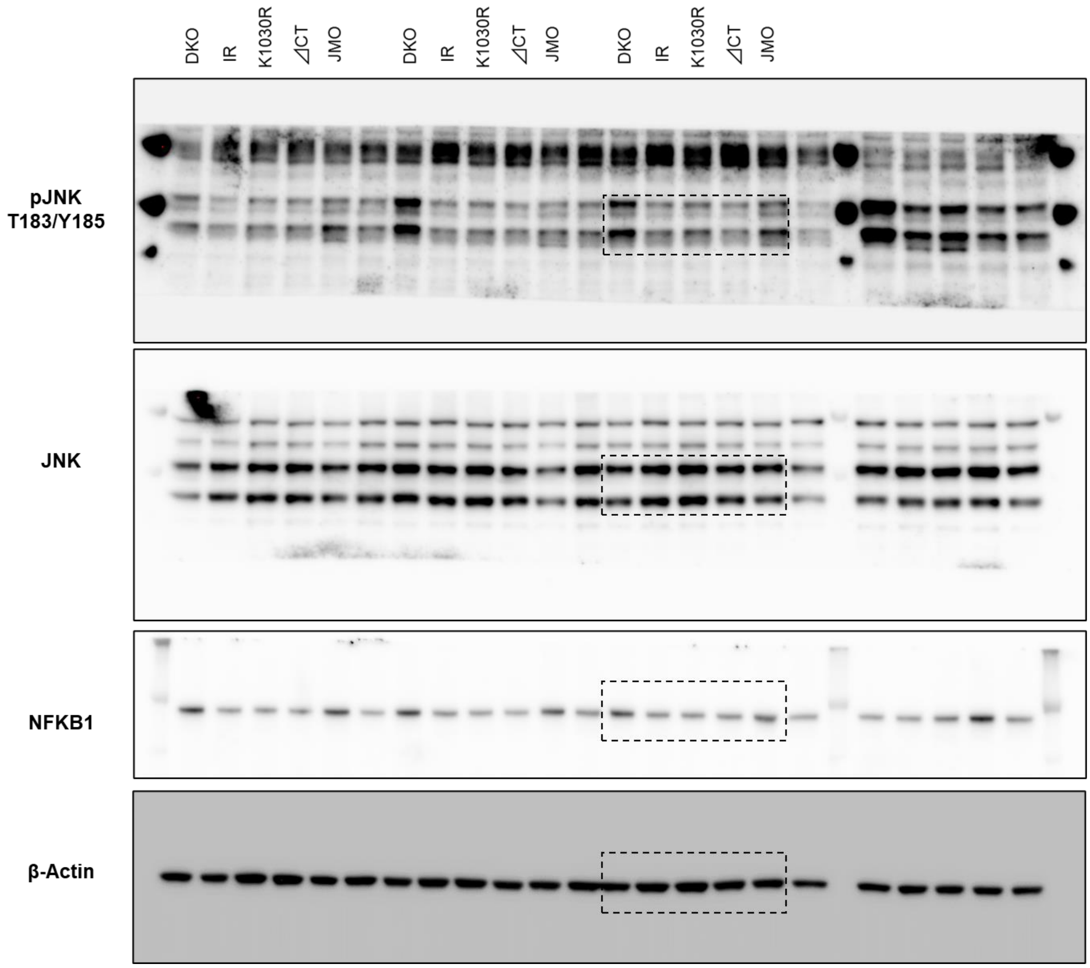

Original blots related to Supplementary Figure 7b

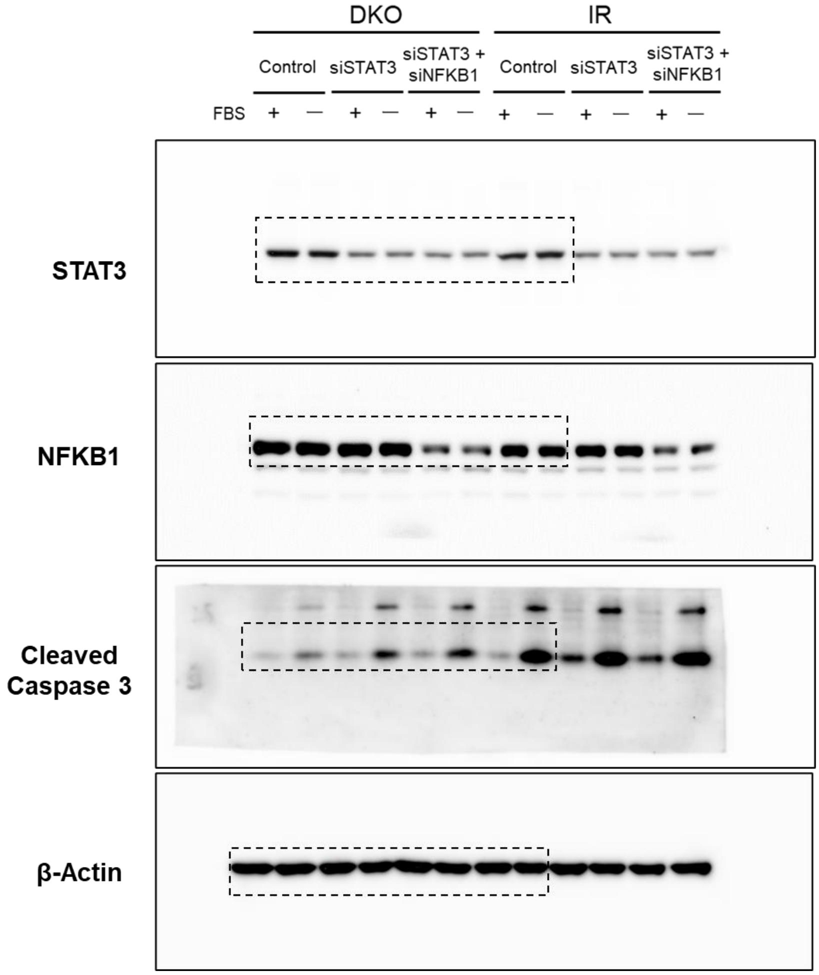

Original blots related to Supplementary Figure 7c

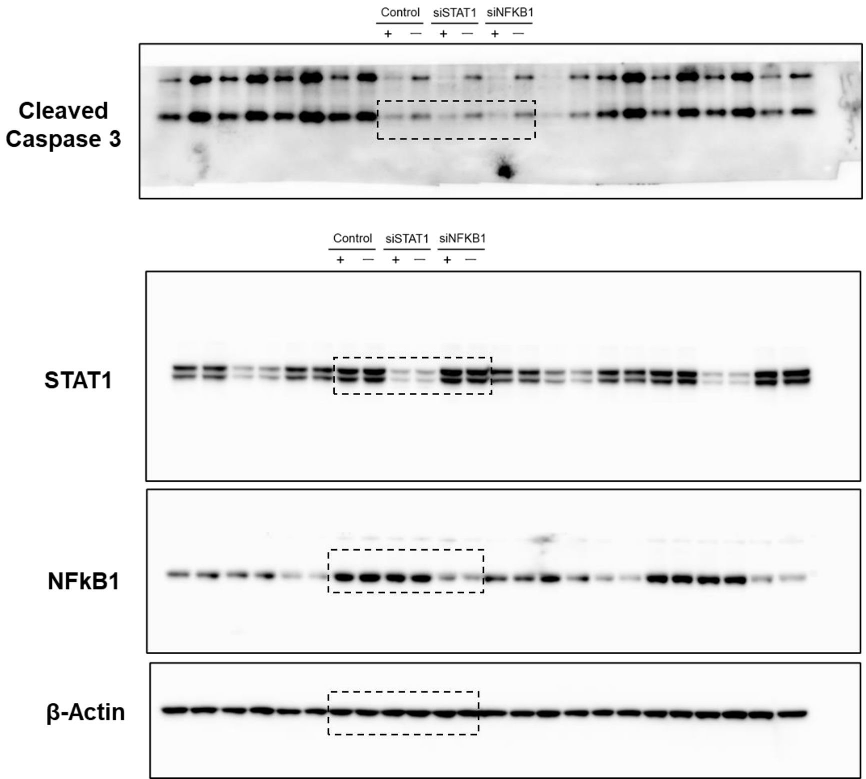

Original blots related to Supplementary Figure 8a

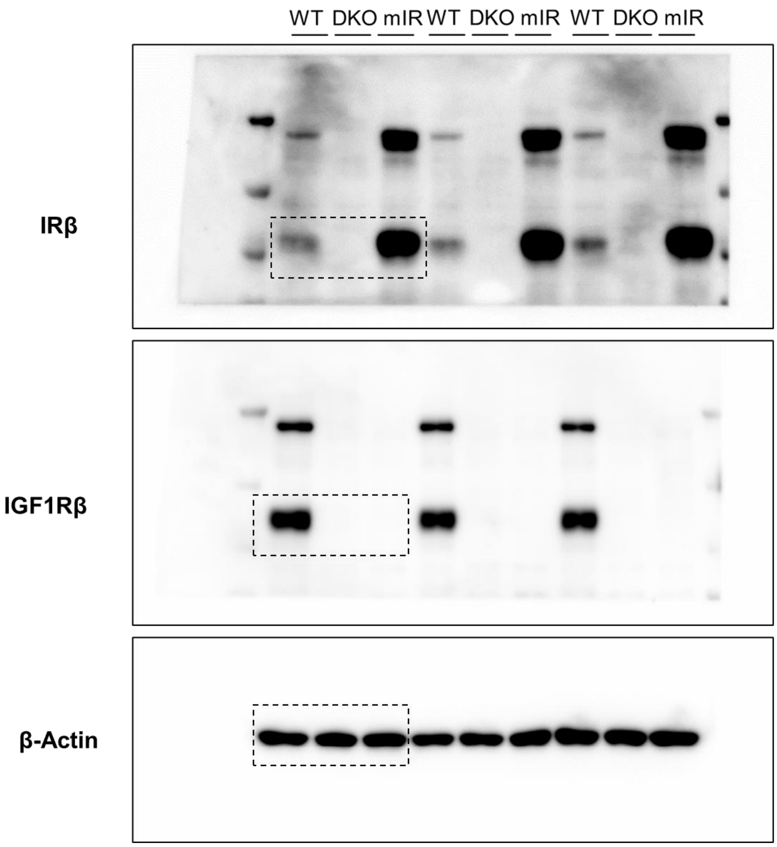

Original blots related to Supplementary Figure 8b

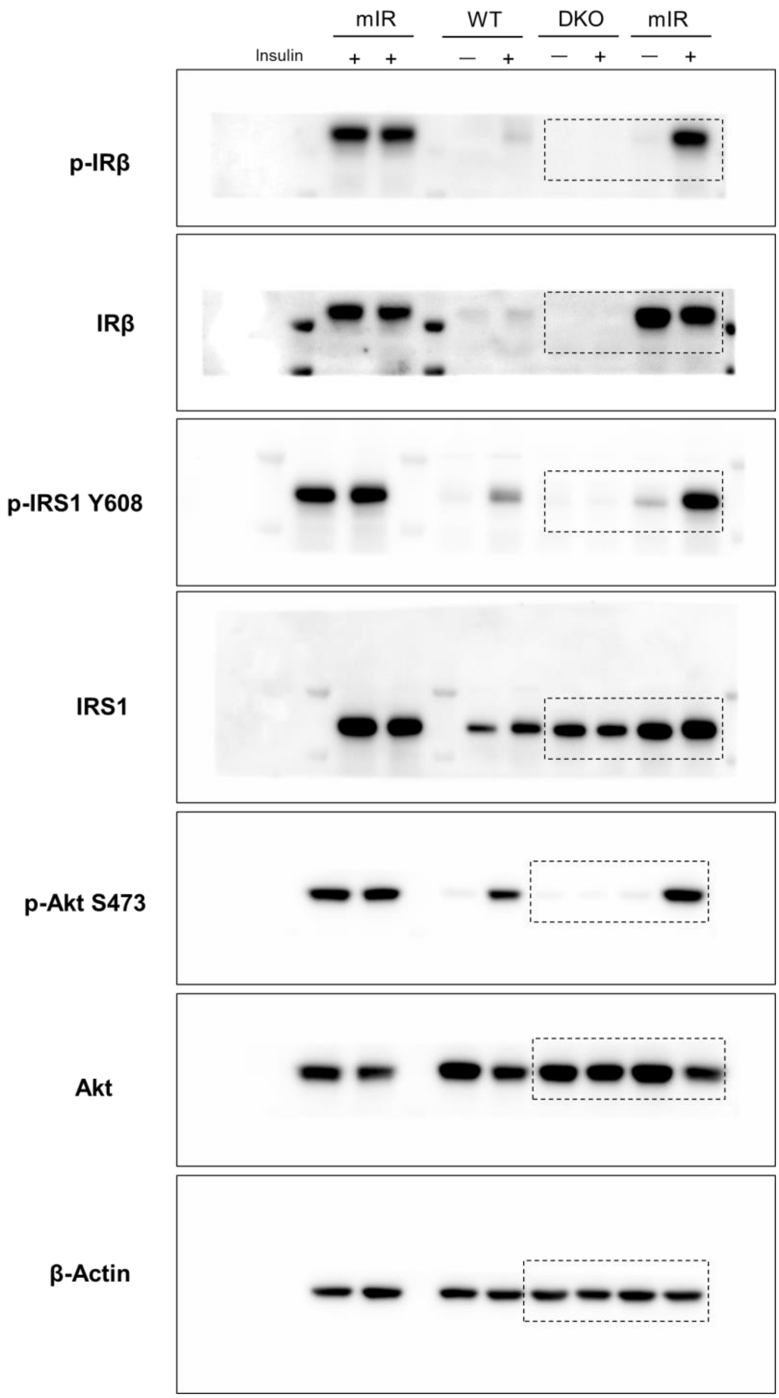

Original blots related to Supplementary Figure 8c

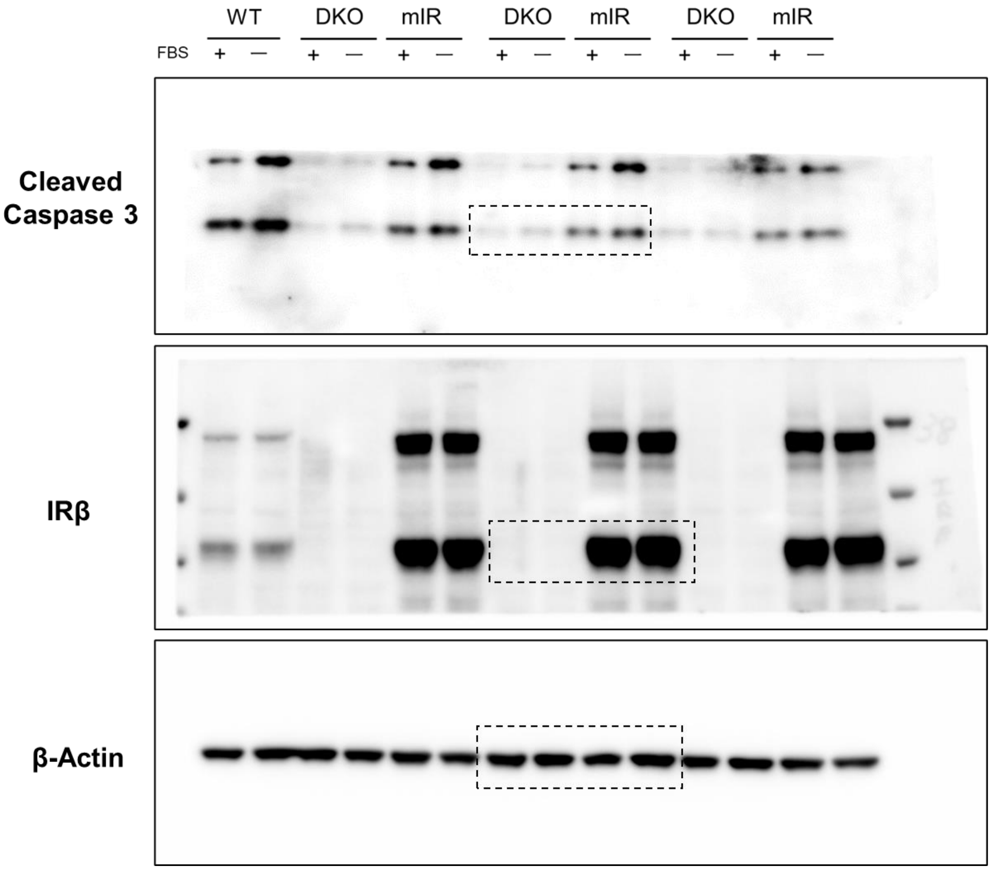

Supplement: Supplementary file 8 — Source Data [file 41467_2022_35693_MOESM8_ESM.zip › Source Data file/Source Data Blots.pdf]
